# A sulfated polysaccharide of Ecklonia cava inhibits the growth of colon cancer cells by inducing apoptosis

**DOI:** 10.17179/excli2014-676

**Published:** 2015-02-24

**Authors:** Ginnae Ahn, WonWon Lee, Kil-Nam Kim, Ji-Hyeok Lee, Soo-Jin Heo, Nalae Kang, Seung-Hong Lee, Chang-Bum Ahn, You-Jin Jeon

**Affiliations:** 1 Department of Marine Bio-Food Science, Chonnam National University, Yeosu 550-749, Republic of Korea; 2 Department of Marine Life Science, Jeju National University, Jeju 690-756, Republic of Korea; 3 Jeju center, Korea Basic Science Institute (KBSI), Jeju 690-140, Republic of Korea; 4 Global Bioresources Research Center, Korea Institute of Ocean Science and Technology, Ansan 426-744, Republic of Korea; 5 Division of Food Bioscience and Korea Nokyong Research Center, Konkuk University, Chungju 380-701, Republic of Korea; 6 Division of Food and Nutrition, Chonnam National University, Gwangju 550-757, Republic of Korea

**Keywords:** Ecklonia cava, sulfated polysaccharides, enzyme-assisted extraction, apoptosis, murine colon cancer cells (CT-26 cells)

## Abstract

We investigated anticancer effects of the crude polysaccharides (CPs) isolated from *Ecklonia cava* enzymatic extracts using AMG, Viscozyme, Protamex, and Alcalase enzyme against a colon cancer cell line, CT26 cells. Among them, the CP of Protamex extract (PCP) contained the highest fucose and sulfated group contents and showed the highest growth inhibitory effect against CT-26 cells. In addition, PCP dose-dependently increased the formation of apoptotic body and the percentage of Sub-G_1_ DNA contents. Also, PCP activated caspase 9 and PARP as regulating the expressions of Bax and Bcl-2. Moreover, PPP2, a fraction purified from PCP showed the highest growth inhibitory effect against CT 26 cells with the increased fucose and sulfated group contents. The results demonstrate that the isolated SP containing plentiful fucose and sulfated group contents has the anticancer effect on colon cancer cells via regulation of Bcl-2/Bax signal pathway.

## Introduction

Seaweeds consisted of brown (Phaeophyta), red (Rhodophyta) and green (Chlorophyta), divided according to the dominant pigment, respectively, xanthophylls, fucoxanthin, phycoerythrin and chlorophyll a and b. They are a widely available source of biomass, considering that over two million tons are either harvested from the oceans or cultured annually for food or phycocolloid production. For few years, various seaweeds have been reported to contain diverse classes of biologically active compounds such as polysaccharides and polyphenols which are useful in the food and pharmaceutical industries (Ahn et al., 2008[2], 2011[3]; Kang et al., 2004[19], 2005[20], [21]). Many researchers have studied about their various biological effects including anticancer, anticoagulant, and anti-inflammatory effects and their major active compounds for few years (Athukorala et al., 2006[6], 2009[5]; Ahn et al., 2008[1]; Hu et al., 2010[17]; Kim et al., 2010[24], [25]; Heo et al., 2010[16]). Among them, algal polysaccharides have been interested by many researchers and reported free radical scavenging, antioxidant, anticoagulant, anticancer, and immunomodulation effects (Zhang et al., 2008[46]; Ahn et al., 2007[2], 2008[1]; Athukorala et al., 2006[6], 2009[5]; Matsuda et al., 2003[35]; Xu et al., 2009[44]). Recently, a brown alga* Ecklonia cava* (*E. cava*) has been also receiving the attention by many researchers due to its beneficial biological effects such as oxygen-radical scavenging (Kang et al., 2004[19]; Ahn et al., 2007[2], 2008[1]), bactericidal effect (Nagayama et al., 2002[38]), antiplasmin inhibiting (Fukuyama et al., 1989[13]), antimutagenesis (Lee et al., 1998[30]). Additionally, the previous studies have reported that its plentiful polysaccharides and small quantities of phenolic compounds led to the beneficial anticancer and antioxidant effects (Athukorala et al., 2009[5]; Ahn et al., 2008[1]; Kang et al, 2005[21], [22]). Also, Athukorala et al. (2009[5]) has reported that the sulfated polysaccharide isolated from an enzymatic extract of *E. cava* inhibited the growth of leukemia cell lines as inducing apoptosis. However, there are no reports about the anticancer effects of the sulfated polysaccharides isolated from enzymatic extracts of *E. cava* on other cancer cell lines such as colon carcinoma, breast cancer, and melanoma cell lines and its biological mechanism. 

Normally, cancer known as a disease manifested by uncontrolled cell growth that presents over 100 distinct clinical pathologies is the largest single cause of death in both men and women, claiming over 6 million lives each year in the world (Kim et al., 2006[26]; Kufe et al., 2003[28]). So, in the last few decades, many anticancer drugs such as chemotherapeutic agents have been developed and used for therapy of cancer patients. However, the use of chemotherapeutic agents for therapy of cancer patients have been unfortunately limited due to their toxicity on normal dividing cell populations resulting in adverse side effects. So, it is important to study the anticancer capacities of natural compounds for the development of anticancer drugs without side effects.

Therefore, in this study, we indicate that a crude polysaccharide isolated from enzymatic extracts of *E. cava* (CPs) contains the plentiful fucose and sulfated group contents and has an anticancer capacity against a colon cancer cell line, CT-26 cells by causing the apoptosis via the Bcl-2/Bax signaling pathway. 

## Materials and Methods

### Chemicals

RPMI-1640 medium, fetal bovine serum (FBS), penicillin–streptomycin, phosphate buffer saline (PBS) and trypsin–EDTA were purchased from Gibco/BRL (Burlington, Ont, Canada). 3-(4,5-Dimethylthiazol-2-yl)-2,5-diphenyltetrazolium bromide (MTT), Ribonuclease A, propidium iodide (PI), ethidium bromide (EtBr), dimethyl sulfoxide (DMSO), and Hoechst 33342 were purchased from Sigma (St. Louis, MO, USA). Antibodies against Bax, Bcl-2, caspase-9, cleaved PARP, and ß-actin were purchased from Cell Signaling Technology (Bedford, Massachusetts, USA). 

### Preparation of enzymatic extracts from E. cava 

The marine alga *E. cava* was collected along the coast of Jeju Island, Korea, between October 2007 and March 2008. To remove salt, epiphytes, and sand attached to the surface, the samples were washed three times with tap water and maintained in a refrigerator at -20 °C. The frozen samples were freeze-dried and homogenized with a grinder for extraction. The enzymatic extracts were prepared following the method developed by Heo et al. (2005[15]). Each one gram of the powdered E. cava were homogenized with 100 mL of distilled water (from pH 4.5 and pH 8.0) and 100 µg or 100 µL of carbohydrases (AMG and Viscozyme) or proteases (Protamex and Alcalase) (Novo Nordisk, Bagsvaerd, Denmark). The reactions were conducted at the property temperature (from 40 °C to 60 °C) for 12 h. Afterward, the digests were boiled for 10 min at 100 °C to inactivate the enzymes and then any unhydrolyzed residues were removed by centrifugation (for 20 min and at 3500 rpm). Finally, the 4 enzymatic extracts obtained after filtration of the supernatants were adjusted to pH 7.0 then stored for use in experiments. 

### Isolation of crude polysaccharides from the enzymatic extracts and the aqueous extract of E. cava (CPs)

Normally, the precipitation technique by ethanol treatment has used to isolate crude polysaccharides from the aqueous extracts (Ahn et al., 2007[2]; Athukorala at al., 2009[5]). Here, the 4 enzymatic extracts prepared from *E. cava* were resolved in 750 mL of distilled water and mixed well with 1.5 L of 99.5 % ethanol, respectively. Then, the mixtures were allowed to stand for 24 h at room temperature and the crude polysaccharide fractions were collected by centrifugation at 20000 rpm for 20 min at 4 °C (Kuda et al., 2002[27]; Matsubara, 2004)[34]. The obtained crude polysaccharides (CPs) were freeze-dried and used for next experiments.

### Isolation of purified polysaccharides (PPP1 and PPP2) from Protamex extract (PCP) by Anion-exchange chromatography 

PCP sample (5 g) was applied to a DEAE-cellulose column (17 cm × 2.5 cm) equilibrated in 50 mM sodium acetate (pH 5.0) and washed with the same buffer containing 0.2 M NaCl (Kang et al. 2011[22]). Elution was carried out at a flow rate 15 mL/h with a linear gradient of 0.2 ~ 0.8 M NaCl containing 50 mM sodium acetate (pH 5.0). The collected two fractions, PPP1 and PPP2 (5 mL) were concentrated by rotary evaporator, dialyzed and freeze-dried for cell growth inhibitory assay.

### Analysis of chemical composition, monosaccharides and sulfate group contents

The chemical compositions of samples were identified by measuring the contents of moisture, proteins, and/or carbohydrates according to the Association of Official Analytical Chemists (AOAC) method. To analyze monosaccharide contents of CPs, the fractions were hydrolyzed in a sealed glass tube with 2 M trifluoroacetic acid for 4 h at 100°C and digested with 6 N of HCl for 4 h. Then, the each samples were applied separately to CarboPacc PA1 (4.5 × 250 mm, Dionex, Sunnyvale, CA, USA) with a CarboPac PA1 cartridge (4.5 × 50 mm) column to analyze neutral and amino sugars, respectively. The columns were eluted by using 16 mM of NaOH at 1.0 mL/min of flow rate. Monosaccharide contents of the samples such as fucose, galactose, and xylose were detected by using a ED50 Dionex electrochemical detector (Dionex), and the data were analyzed by Peack Net software (Dionex). Additionally, their sulfate contents were analyzed in accordance to the BaCl2/galation method (Saito et al., 1968[40]). The preparation’s components were expressed as w/w (g/100g) or %, respectively. 

### Cell culture 

FM3A cell (mouse mammary carcinoma cell line) originated from the mammary gland of the C3H/He mouse was offered by Dr. Won-Suk Kim (BINEX Co., Ltd.). B16F10 (mouse melanoma cell line) and CT26 (mouse colon carcinoma cell line) cells were purchased from the KCLB (Korean Cell Line Bank, Seoul, Korea). These cells were grown on RPMI-1640 medium supplemented with 10 % (v/v) heat-inactivated FBS, penicillin (100 U/mL) and streptomycin (100 µg/mL). Cultures were maintained at 37 °C in a 5 % CO_2_ incubator.

### Cell growth inhibitory assay 

To identify whether the 5 CPs and the purified two fractions can inhibit the growth of tumor cells, a colorimetric MTT assay was performed. Normally, the colorimetric MTT assay is dependent on the conversion of yellow tetrazolium bromide to its purple formazan derivative by mitochondrial succinate dehydrogenase in viable cells (Mossmann, 1983[37]). Briefly, three attached cells (FM3A, B16F10, and/or CT26 cells) were seeded in 96 well plates at a concentration of 0.5 × 10^4^ cells/well. After 16 h, the cells were treated with the isolated five CPs and/or two fractions (18.8, 37.5, and/or 75.0 µg/mL). The cells were then incubated for the additional 24, 48, and/or 72 h at 37 °C. MTT stock solution (50 µL; 2 mg/mL in PBS) was added to each well and more incubated for 4 h. And then, the plates were centrifuged for 10 min at 2000 rpm and the supernatants were aspirated. The formazan crystals were dissolved by adding DMSO and their absorbance was measured at a wavelength of 540 nm by ELISA reader (Tecan, Männedorf, Switzerland). The data were expressed as mean percentages of the viable cells versus the respective control. 

### Nuclear staining with Hoechst 33342 

To identify whether PCP can affect to morphologic changes of CT26 cells, we performed Hoechst 33342 staining assay. Generally, the nuclear morphology of cells has evaluated by using a DNA-specific fluorescent dye, Hoechst 33342 staining nuclei of cells. According to its feature, cells with homogeneously stained nuclei were considered viable, whereas the presence of chromatin condensation and/or fragmentation was indicative of apoptosis (Gschwind and Huber 1995[14]; Lizard et al. 1995[33]). And, CT26 cells were seeded in 24 well culture plates at a concentration of 4 × 10^5^ cells/well. After 16 h, the cells were treated with various concentrations of PCP (18.8, 37.5, and 75.0 µg/mL) and incubated for an additional 72 h. Then, 10 µg/mL of Hoechst 33342 was added to the each well and the plates were incubated for an additional 10 min at 37 °C. The stained cells were observed under a fluorescence microscope equipped with a CoolSNAP-Pro color digital camera in order to determine the degree of nuclear condensation. 

### Propidium iodide (PI) staining assay 

Flow cytometry analysis using a propidium iodide (PI) dye that can stain DNA has been performed to determine the proportion of apoptotic sub-G_1_ hypodiploid cells known as a feature of apoptosis (Nicoletti et al., 1991[39]). CT26 cells were seeded in 6 well culture plates at a concentration of 1 × 10^6^ cells/well. After 16 h, the cells were treated with various concentrations of PCP (18.8, 37.5, and 75.0 µg/mL). After incubation of an additional 72 h, the cells were harvested and fixed in 1 mL of cold 70 % ethanol for 30 min at 4 °C. Then, the cells were washed twice with buffer 1 consisted of PBS containing 2 mM-EDTA and incubated in 1 mL of buffer 2 (buffer 1 containing 10 µg of PI and 50 µg of RNase A) for 30 min at 37 °C in darkness. To determine the effects of the polysaccharide on changes in the percentage of cell distribution at each cell cycle phase, ten thousand cells were analyzed using a BD FACSCalibur^TM^ flow cytometer (BD Biosciences, San Jose, CA, U.S.A.) and the and CellQuest software. 

### Western blot analysis 

Effect of PCP on the expression of apoptosis-related molecules such as Bax, Bcl-2, cleaved PARP, and caspase-9 was assessed by Western blot analysis. CT26 cells (2 × 10^5^ cells/mL) were seeded in 100 mm culture dishes and cultured for 16 h. Then, the cells were treated with three concentrations of PCP (18.8, 37.5, and 75.0 µg/mL). After incubation of 72 h, the cytoplasmic proteins were extracted from the cells by using lysis buffer (50 mM/L Tris-HCl (pH 7.4), 150 mM/L NaCl, 1 % Triton X-100, 0.1 % sodium dodecyl sulfate (SDS) and 1 mM/L EDTA). Cytoplasmic (50 mg/well) preparation was loaded into SDS-PAGE (SDS-polyacrylamide gels) and electrophoresed under denaturing conditions. Subsequently, proteins were electro-transferred onto nitrocellulose transfer membrane. After blocking with 5 % nonfat milk for 2 h, blots were incubated with primary antibodies such as Bax (1:1000 dilution), Bcl-2 (1:1000 dilution), cleaved PARP (1:1000 dilution), caspase-9 (1:1000 dilution), or ß-actin (1:5000 dilution) antibodies for 60 min followed by incubation with horseradish peroxidase (HRP)-conjugated anti-mouse or anti-rabbit immunoglobulin (Ig) G (Cell Signaling Technology Inc.) for 60 min. Visualization was achieved by using X-ray film and chemiluminescence reagents. 

### Statistical analysis 

Results are reported as standard deviation (SD). For statistical analysis, normally data were analyzed by one-way ANOVA (analysis of variance). Where appropriate, data were compared using the unpaired Student’s t-test. Analysis results were considered to be statistically significant if *p *< 0.05.

## Results

### Application of the enzyme-assisted extraction process improved the extraction efficiency of crude polysaccharides (CPs) components

To improve extraction efficiency of polysaccharide contents from *E. cava*, we performed the enzymatic-assisted extraction by using four enzymes (AMG, Viscozyme, Protamex, and Alcalase). As demonstrated in Table 1(Tab. 1), the application of enzyme-assisted extraction technique improved the most of extraction yields (from 32.4 ± 0.1 % to 36.1 ± 0.4 %) from *E. cava*, compared to its aqueous extract (28.0 ± 0.6 %). Also, the extraction yields (%) of crude polysaccharides (CPs) isolated from four enzymatic extracts prepared by using carbohydrases and proteases (from 5.1 ± 0.3 % to 15.6 ± 0.4 %) were higher than that of ACP (3.9 ± 0.2 %) (Table 2(Tab. 2)). In particular, CPs isolated from two enzymatic extracts prepared by using carbohydrases (AMGCP and VCP) led to the higher extraction yields, compared to the others (PCP and ALCP). 

### PCP showed the highest fucose and sulfated group contents

Next, we analyzed the contents of CPs isolated from four enzymatic extracts and one aqueous extract by measuring the chemical composition such as moisture, protein, and carbohydrate and the contents of sulfate group and monosaccharides such as fucose, galactose and etc. As indicated in Table 2(Tab. 2), the moisture and protein contents of CPs isolated from four enzymatic extracts were similar to those of ACP. However, the contents of carbohydrate in CPs isolated from 4 enzymatic extracts were weakly increased, compared to ACP. Especially, PCP showed the highest carbohydrate contents (51.3 ± 0.2 %) among the others. Also, the results showed that CPs isolated from the four enzymatic extracts of *E. cava* are composed mainly fucose and galactose with small quantities of other monosaccharides such as mannose, glucose, and xylose and sulfate group. Interestingly, the CPs of two enzymatic extracts prepared by proteases (PCP, ALCP, and VCP) showed the higher contents of fucose, compared to those of the other extracts (ACP and AMGCP) (71.2 ± 0.5 %, 71.2 ± 0.3 %, and 62.2 ± 0.2 % vs 57.8 ± 0.4 % and 57.1 ± 0.2 %, respectively) (Table 2(Tab. 2)). But, the CPs of enzymatic extracts showed the higher sulfated group contents. 

### PCP induced the highest growth inhibitory effect against CT 26 cells

**Figure 1:**
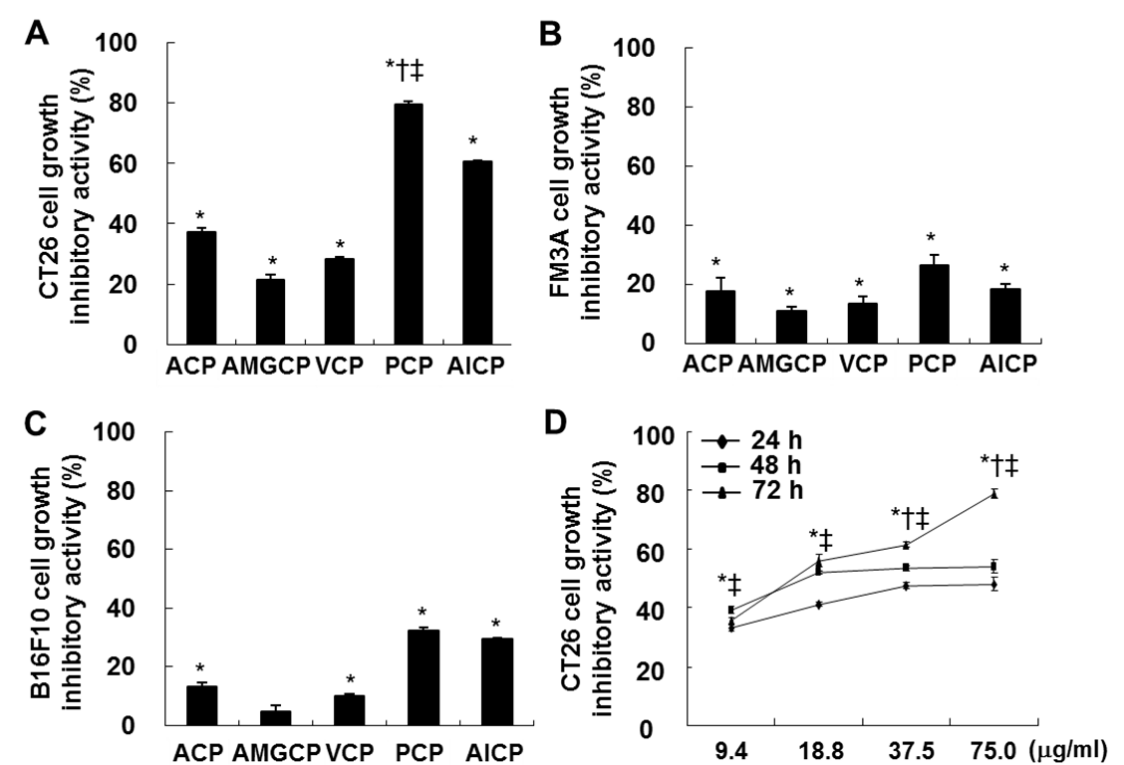
Effects of CPs against the growth inhibition in FM3A (A), B16F10 (B), and CT 26 (C and D) cells. The cells (0.5 × 10^4^ cells/well) incubated with CPs (9.4, 18.8, 37.5, and/or 75.0 µg/mL) for 24 h, 48 h, and/or 72 h were used for MTT assay. The results are representative of three separate experiments (n = 3). Significance was expressed as *; *P* < 0.05 vs. non-treated control cells, †; *P* < 0.05 vs. ACP-treated cells, and ‡; *P *< 0.05 vs. AlCP-treated cells for 72 h.

To identify growth inhibitory effects of the isolated CPs in FM3A, B16F10, and CT26 cells, MTT assay was performed. As indicated in Figure 1A(Fig. 1), 1B(Fig. 1), and 1C(Fig. 1), the most of CPs isolated from the aqueous and enzymatic extracts showed the inhibitory activities against the growth of FM3A, B16F10, and CT26 cells at the concentration of 75.0 µg/mL, compared to the non-treated control cells. As shown in Figure 1A(Fig. 1) and 1B(Fig. 1), the all tested CPs slightly inhibited the growth of FM3A and B16F10 cells. However, CPs iso-lated from 4 enzymatic extracts and ACP led to the various growth inhibitory effects against CT26 cells, compared to the non-treated control cells (*; P < 0.05) (Figure 1A(Fig. 1)). In particular, PCP showed the highest growth inhibitory effect than the others (79.5 ± 1.1 %) (*;* P* < 0.05 vs. non-treated control cells, †; *P* < 0.05 vs. ACP-treated cells, and ‡; *P* < 0.05 vs. AlCP-treated cells). Moreover, PCP significantly inhibited the growth of CT26 cells at all incubation times (24, 48, and 72 h) (*; *P *< 0.05 vs. non-treated control, †; *P* < 0.05 vs. PCP-treated cells at 48 h, ‡; *P* < 0.05 vs. PCP-treated cells at 72 h) (Figure 1D(Fig. 1)). In particular, the growth of CT26 cells was dose-dependently inhibited by PCP at all the concentrations (from 9.4 µg/mL to 75.0 µg/mL) at 72 h, compared to the non-treated control cells. 

### PCP dose-dependently increased the formation of apoptotic body and the percentage of apoptotic sub-G1 DNA content in CT 26 cells

**Figure 2:**
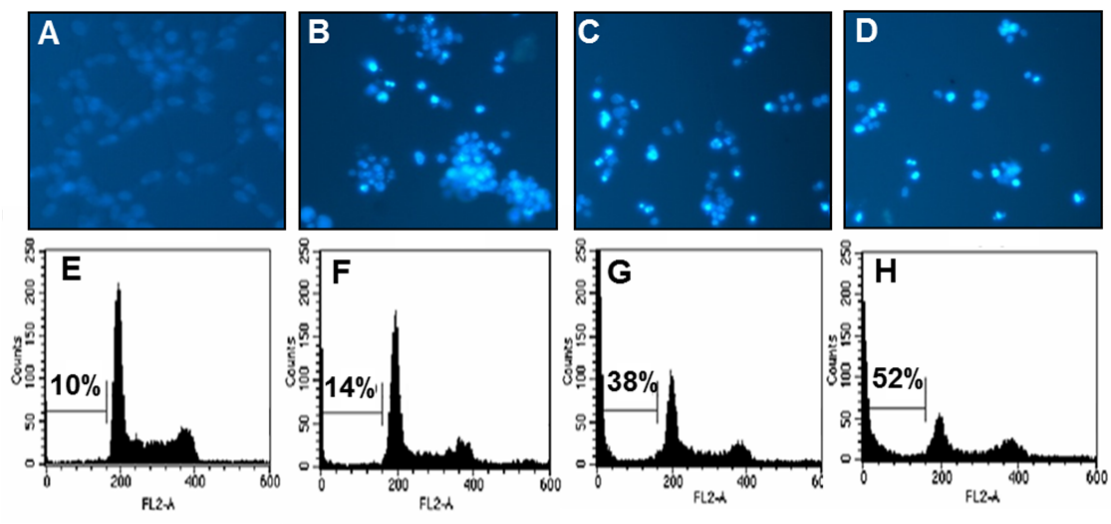
Effects of PCP on the induction of apoptotic bodies (A-D) and the percentage of sub-G_1_ DNA contents (E-H) in CT 26 cells. The cells were incubated with PCP (18.8, 37.5, and 75.0 µg/mL) for 72 h. (A-D) Apoptotic bodies were stained with Hoechst 33342 solution and then observed under a fluorescent microscope using a blue filter. (E-H) The cells were stained with PI and analyzed via flow cytometry. The results are representative of three separate experiments (n = 3). A and E; the non-treated control cells, B and F; the cells treated with 18.8 µg/mL of PCP, C and G; the cells treated with 37.5 µg/mL of PCP, D and H; the cells treated with 75.0 µg/mL of PCP.

We used the microscopic detection using Hoechst 33342 staining to check whether PCP induces the apoptotic fragments known as a characteristic of apoptosis in CT 26 cells. As shown in Figures 2A(Fig. 2), 2B(Fig. 2), 2C(Fig. 2), and 2D(Fig. 2), the microscopic images showed that PCP markedly increased the formation of nuclear fragmentation such as the apoptotic bodies in a dose-dependent manner, compared to that of non-treated control cells. In particular, the treatment of PCP (75.0 µg/mL) markedly caused the morphological changes and the formation of apoptotic fragments in CT 26 cells comparing to the control cells. Moreover, PCP dose-dependently caused the changes of cell cycle phases as markedly increasing apoptotic sub-G_1_ DNA content in CT 26 cells, compared to the non-treated control cells (Figures 2E(Fig. 2), 2F(Fig. 2), 2G(Fig. 2), and 2H(Fig. 2)). Especially, 75.0 µg/mL of PCP significantly increased the percentage of apoptotic sub-G_1_ DNA content up to 52 ± 2.1 %, comparing to the non-treated control cells (10 ± 1.2 %). These results are supported by previous study reported that during apoptosis of the cancer cells, the DNA of individual cells is appeared in a hypo diploid sub-G_0/1_-peak as a consequence of partial DNA loss (Ehemann et al., 2003[12]). Also, this suggests that PCP induced the apoptosis of CT 26 cells and it affected to its anti-proliferative effects.

### PCP dose-dependently activated PARP and caspase 9 by regulating Bcl-2/Bax signaling

**Figure 3:**
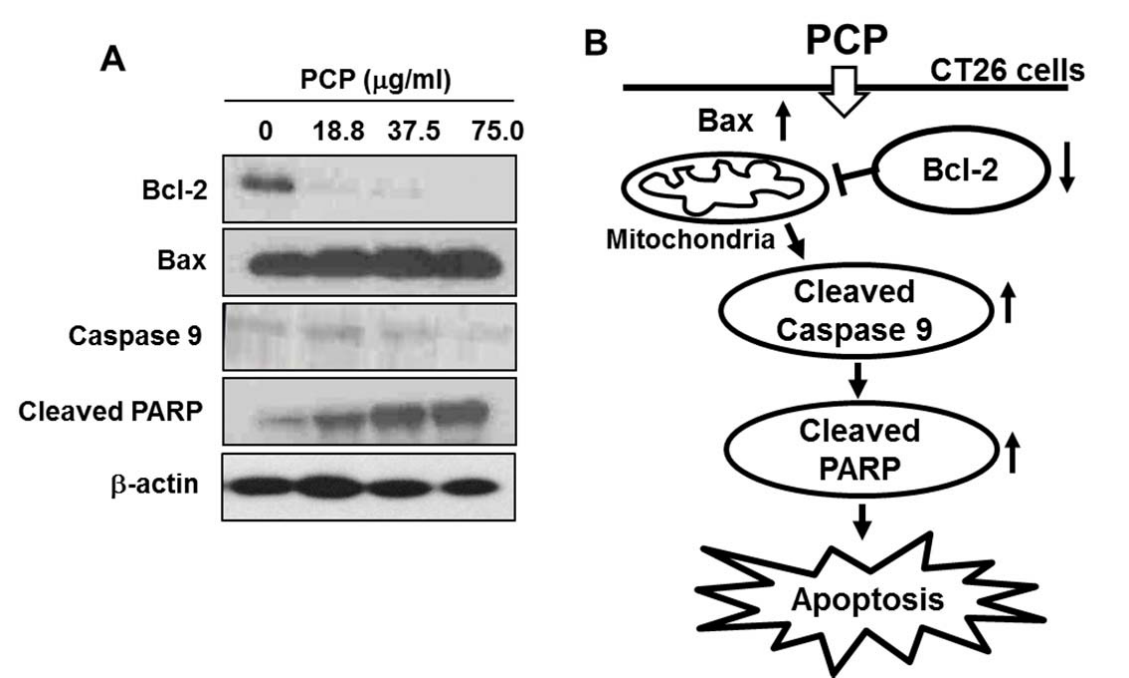
Effect of PCP on the expressions of apoptosis-related proteins such as Bcl-2, Bax, caspase 9, and PARP in CT 26 cells. The cells were incubated with PCP (18.8, 37.5, and 75.0 µg/mL) for 72 h. Equal amounts of cell lysates (50 µg) were resolved via SDS-PAGE, transferred to nitrocellulose, and probed with specific antibodies, as indicated in Materials and Methods part. ß-actin was used as internal control. The results are representative of three separate experiments (n = 3).

In Figure 3(Fig. 3), PCP markedly decreased the expression of Bcl-2, whereas slightly increased the expression of Bax, compared to non-treated control cells. In addition, the treatment of PCP considerably increased the cleavage of PARP and caspase 9 in a dose-dependent manner. 

### PPP2, a fraction purified from PCP by anion-exchange chromatography markedly inhibited the growth of CT 26 cells

**Figure 4:**
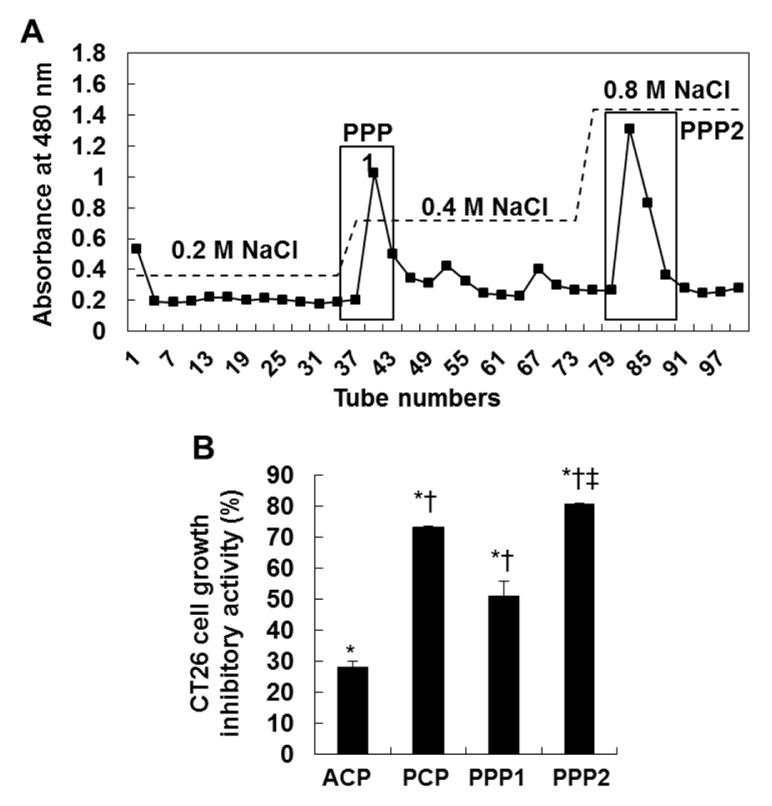
DEAE-cellulose chromatogram of the purified two fractions (PPP1 and PPP2) (A) and the growth inhibitory effect of ACP, PCP, PPP1 and PPP2 against CT 26 cells (B). The cells (0.5 × 104 cells/well) incubated with CPs (75.0 µg/mL) for 72 h were used for MTT assay. The results are representative of three separate experiments (n = 3). Significance was expressed as *; *P* < 0.05 vs. untreated cells, †; *P* < 0.05 vs. ACP-treated cells, and ‡; *P* < 0.05 vs. PCP-treated cells for 72 h.

Next, to isolate purified sulfated polysaccharides from PCP, we performed anion-exchange chromatography and purified two fractions from PCP (PPP1 and PPP2) (Figure 4A(Fig. 4)). Then, we evaluated their anti-proliferative effects against CT 26 cells by using MTT assay. The results showed that PCP and its two fractions (PPP1 and PPP2) (75.0 µg/mL) showed the higher growth inhibitory effects against CT 26 cells for 72 h, compared to those of the non-treated control cells and ACP-treated cells (*; *P* < 0.05 vs. non-treated cells, †; *P* < 0.05 vs. ACP-treated cells, and ‡; *P* < 0.05 vs. PCP-treated cells). In particular, PPP2 showed the highest growth inhibitory effect against CT 26 cells among the others. But the effect of PPP1 was lower than that of PCP, although it was higher than that of ACP. Interestingly, PPP2 showed the increased fucose and sulfated group contents with the carbohydrate contents, compared to both PPP1 and PCP (75.1 ± 2.1 % and 23.4 ± 0.4 % in PPP2 vs 65.1 ± 0.7 % and 14.6 ± 0.6 % in PPP1 and 71.2 ± 0.5 % and 17. 3 ± 3.0 % in PCP, respectively) (Table 2(Tab. 2) and Table 3). 

## Discussion

Normally,* E. cava* contains large amount of carbohydrate (> 60 %) and small amount of proteins and polyphenol components. Among these components, the polysaccharides included in carbohydrate contents have been recently received the attention by many researchers who study various biological activities such as anticancer on a human leukemia cancer cell line (U937 cells) and anticoagulant activities (Athukorala et al., 2006[6], 2009[5]). In addition, previous studies have been reported that many kinds of polysaccharides isolated from plants and seaweeds have anticancer effects as caused cellular damages by inducing apoptosis in various cancer cell lines for few years (Xu et al., 2009[44]; Teruya et al., 2007[42]). In particular, our previous study indicated the anticancer effect of a purified sulfated polysaccharide from* E. cava* on human leukemia cancer cells (Athukorala et al., 2009[5]). Here we wanted to more reveal anticancer effects of polysaccharides from* E. cava* against other cancer cells such as FM3A (mouse mammary carcinoma cell line), B16F10 (mouse melanoma cell line), and CT26 (mouse colon carcinoma cell line) cells. Normally, the enzyme-assisted extraction process using the hydrolysis capacities of enzymes can improve nutritional and functional properties of foods as increasing the extraction of active compounds such as polysaccharides and peptides, ingestion rate, and body absorption (Heo et al., 2010[16]; Lee et al., 2011[31]). In this present study, we showed that the application of the enzyme-assisted extraction process improved the extraction efficiency of polysaccharide component. This result indicates that the ennzyme-assisted extraction process using the carbohydrases and proteases affected to increase the extraction efficiency of CP components. Previous studies have reported that fucoidan has been known as a complex sulfated polysaccharide found in the cell walls of several edible brown algae, including *Fucus vesiculosus* (Bilan et al., 2002[8], 2006[7]; Li et al., 2008[32]). The structures and compositions of fucoidan vary among different brown seaweed species, but generally the compound consists primarily of L-fucose and sulfate, along with small quantities of D-galactose, D-mannose, D-xylose, and uronic acid (Bilan et al., 2002[8], 2006[7]; Li et al., 2008[32]). Our previous studies also indicated that *E. cava* has sulfated polysaccharide compounds containing the plentiful fucose and sulfate group contents for biological activities (Athukorala et al., 2006[6], 2009[5]; Ahn et al., 2008[1]). With corresponding to these findings, our result indicates that the isolated CPs can be sulfated polysaccharides which can be regarded as a fucoidan due to their high fucose and sulfate group contents. Also, in our results, PCP contains the higher fucose and sulfate group led to the highest growth inhibitory effect against CT26 cells, although the extraction efficiency did not show the relationship with their anti-proliferative capacities. 

Generally, apoptosis is a form of programmed cell death essential for homeostasis, which is frequently deregulated in human pathologies such as cancer, neurodegenerative diseases or viral infections (Meier et al., 2000[36]; Vaux and Korsmeyer, 1999[43]). As compared to necrosis, apoptotic cells are undergone cellular DNA damages with morphological changes such as the budding and formation of apoptotic bodies but also DNA fragmentation (Sgong and Gruber, 1998[41]; Compton and Cidlowski, 1986[10]). In addition, microscopic detection of structural alterations is the most reliable method to identify apoptotic cells (Kerr et al., 1972[23]). We showed PCP dose-dependent-ly increased the formation of apoptotic body and the percentage of apoptotic sub-G_1_ DNA contents in CT 26 cells. These results are supported by previous study reported that during apoptosis of the cancer cells, the DNA of individual cells is appeared in a hypo diploid sub-G_0/1_-peak as a consequence of partial DNA loss (Ehemann et al., 2003[12]). Also, this suggests that PCP induced the apoptosis of CT 26 cells and it affected to its anti-proliferative effects. Additionally, PCP dose-dependently activated PARP and caspase 9 by regulating Bcl-2/Bax signaling. Bax is essential for death receptor-mediated apoptosis in cancer cells (LeBlanc et al., 2002[29]), whereas the carboxyl-terminal Bcl-2 cleavage product triggers cell death (Cheng et al., 1997[9]). Our previous study have reported that the SP of* E. cava* inhibited the growth of U937 cells, a leukemia cell line as causing DNA damages such as the formation of apoptotic bodies and DNA fragmentation through regulating mitochondrial pathway related with apoptotic molecules such as Bcl-2 and Bax (Athukorala et al., 2009[5]). In addition, Kim et al. (2010[24]) previously reported that fucoidan induced apoptosis in human colon cancer cells, but that the efficacy of fucoidan in inducing apoptosis varies among different types of colon cancer cells. Also, many researchers have noted that fucoidan induces apoptosis via the activation of caspases and the regulation of MAPK including ERK, p38 kinase, and Akt pathway as well as the changes of Bcl-2 and Bax in MCF-7 and HCT-15 cells (Aisa et al., 2005[4]; Hyun et al., 2009[18]; Yamasaki-Miyamoto et al., 2009[45]). In colon cancer cells, fucoidan also induced apoptosis through the activation of caspase 3 and 9 and mitochondria-mediated apoptotic pathways (Kim et al., 2010[24]). In accordance with the letter findings, we indicate that PCP-induced the activation of caspase 9 and PARP via regulation of the Bcl-2/Bax signal pathway contributed to cellular DNA damages such as the formation of apoptotic bodies in nuclei and DNA contents in sub-G_1_ phase in CT26 cells (Figure 3B(Fig. 3)). However, it remains to be investigated whether PCP is related with MAPK signal pathways including ERK and p38 kinase and Akt pathway in CT 26 cells. In the result, we revealed PPP2, a fraction purified from PCP by anion-exchange chromatography markedly inhibited the growth of CT 26 cells. These results indicate that the enhanced cell growth effect was corresponded to the increased contents of fucose and sulfated groups contained in the samples. Also, our previous study suggested the molecular weight of the polysaccharide purified from AMG extract of* E. cava* was approximately 1.381 × 10^6^ Da (Kang et al, 2011[22]). Although the process for the extraction and isolation is little different, we can consider the molecular weight of the purified polysaccharide, PPP2 might be similar to it. From these results, we indicate that the fucose and sulfated group contents can affect to the enhancement of anticancer effects against CT 26 cells. Moreover, PPP2 can be regarded as a SP due to its plentiful contents of fucose and sulfated group and they play important key roles in the anticancer effect against CT 26 cells. 

In conclusion, this present study revealed that the isolated sulfated polysaccharides (PCP and/or PPP2) consisted of plentiful fucose and sulfated group contents have anticancer effects against CT26 colon carcinoma cells via regulation of the Bcl-2/Bax signal pathway. Furthermore, this study suggests that the purified polysaccharide can be regarded as an anticancer fucoidan and contribute to development of natural anticancer drugs or carbohydrate polymer industries. Further study should be conducted in the future to more evaluate the potential mechanism of PPP2 as a colon cancer-preventive agent in experimental animal models and humans.

## Notes

Ginnae Ahn and WonWoo Lee contributed equally to this work.

## Acknowledgements

This work was supported by the Korea Research Foundation Grant funded by the Korean Government (MOEHRD, Basic Research Promotion Fund) (KRF- 2008-F00016) and the Ministry of Agriculture, Food and Rural Affairs (MAFRA), Ministry of Oceans and Fisheries (MOF), Rural Development Administration(RDA) and Korea Forest Service (KFS) (213004-04-2-SB930). 

## Figures and Tables

**Table 1:**
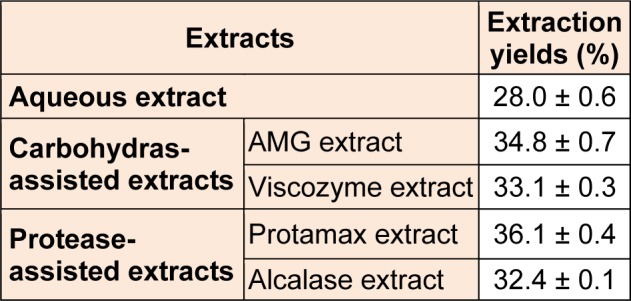
Extraction yields (%) of four enzyme-associated extracts and one aqueous extract from *Ecklonia cava *(*E. cava*)

**Table 2:**
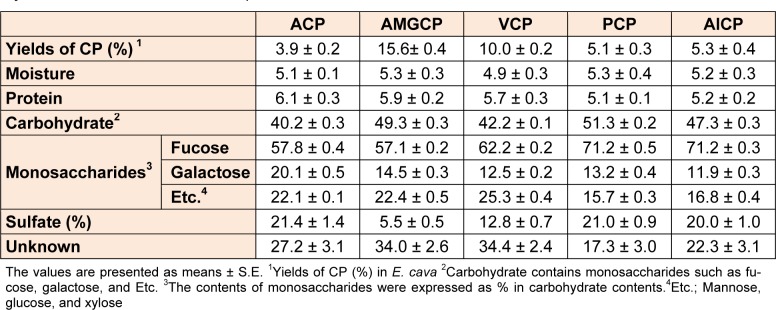
Yields (%) and chemical composition (%) of 5 crude polysaccharides (CPs) isolated from enzyme-associated extracts and aqueous extract of *E. cava*
